# Report of Missed Non-Hodgkin’s Lymphoma Presenting as Pathological Tibial Fracture

**DOI:** 10.7759/cureus.18914

**Published:** 2021-10-20

**Authors:** Ellery Altshuler, Kellen Worhacz

**Affiliations:** 1 Internal Medicine, University of Florida College of Medicine, Gainesville, USA; 2 Orthopedic Surgery, Banner Sports Medical Center, Phoenix, USA

**Keywords:** lymphoma of the bone, missed diagnosis, proximal tibia fracture, non hodgkin's lymphoma, pathological fracture

## Abstract

Pathological fractures usually occur in patients with known malignancies, though pathological fracture may be the first sign of cancer. Malignant pathological fractures most commonly represent metastatic lung, breast, kidney, thyroid, or prostate cancer and typically occur in the spine, pelvis, femur, and humerus. We present the case of a 71-year-old female with an atraumatic tibial fracture in which further imaging was not pursued. Five months later, she was diagnosed with non-Hodgkin’s lymphoma. Pathological proximal tibial fracture is an exceedingly rare presentation of lymphoma; however, diagnostic delay could likely have been avoided if a thorough workup had been pursued. Our case highlights the importance of careful examination of all patients presenting with a low-energy fracture, particularly when constitutional symptoms suggestive of cancer are present. All patients with pathological fractures should receive a thorough physical examination that includes lymph node palpation.

## Introduction

Atraumatic fractures include fractures that arise in the setting of a low-impact mechanism that under ordinary circumstances would not produce fracture [1]. These fractures occur with minimal force due to an underlying condition that has disrupted skeletal physiology [2]. Pathological fractures are usually caused by neoplasms, though fractures due to osteomyelitis and metabolic derangements are also considered pathological [2]. Although cancer has been previously diagnosed in most patients who suffer a pathological fracture, a nontraumatic injury may also be the first sign of metastatic cancer, multiple myeloma, primary bone tumor, or lymphoma.

## Case presentation

**Figure 1:**
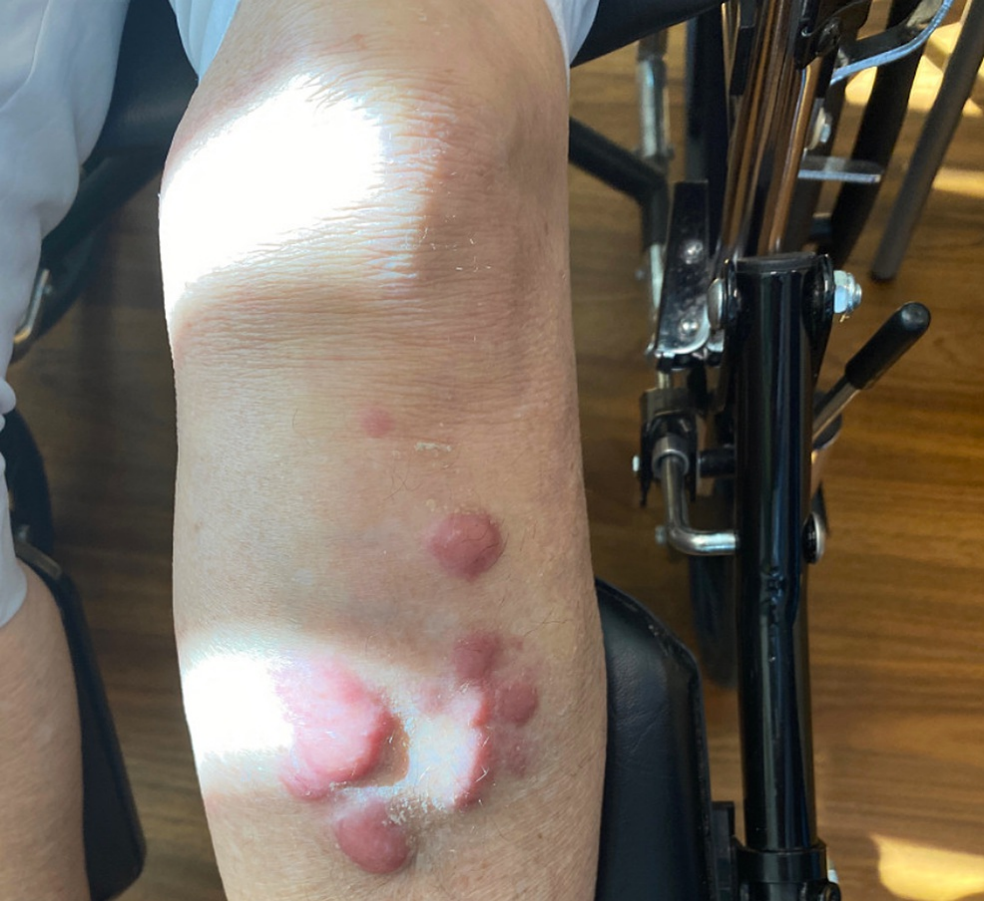
Left leg just prior to biopsy. There is gross deformity and large erythematous skin lesions in the area of the proximal tibial lymphoma.

A 71-year-old female with a past medical history of hypothyroidism presented to the emergency department with pain in her left leg that began after she stepped off a one-foot curb. She described a low-energy mechanism of injury. Radiographs in the emergency department demonstrated a non-displaced, proximal-third left tibia fracture. Additional history included a 20-pound weight loss over the preceding several months. Labs on presentation were unremarkable, with a calcium level of 9.2 mg/dl (reference: 8.4-10.2 mg/dl). She was considered likely to have osteoporosis and was managed conservatively with immobilization of the affected extremity and referred to physical therapy. Despite these measures, her pain worsened, and she became increasingly immobile, eventually requiring the use of a wheelchair. She also noted large erythematous cutaneous nodules developing over her anterior leg in proximity of the original fracture location (Figure 1).

**Figure 2:**
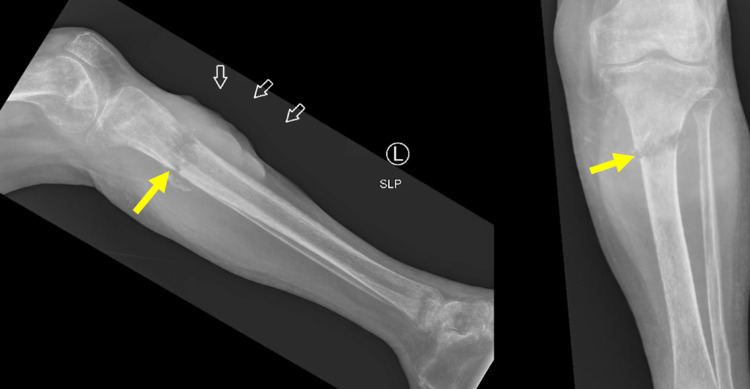
Radiographs from the time of diagnosis of proximal tibial lymphoma, five months after the patient suffered an atraumatic leg injury. The fracture (yellow arrows) appears as a transverse band of lucency with ill-defined margins. A soft tissue mass (white arrows) is also apparent.

Five months after her original injury, repeat radiographs were obtained and showed a nondisplaced pathologic fracture involving the proximal tibial diaphysis with an adjacent soft tissue mass (Figure 2). Notable lab values included a hemoglobin of 8.3 g/dl (reference: 12.0-16.0 g/dl), lactate dehydrogenase of 978 international units/liter (IU/l) (reference: 135-225 IU/l), and a C-reactive protein 86.5 mg/l (reference: 0.0-5.0 mg/l).

**Figure 3:**
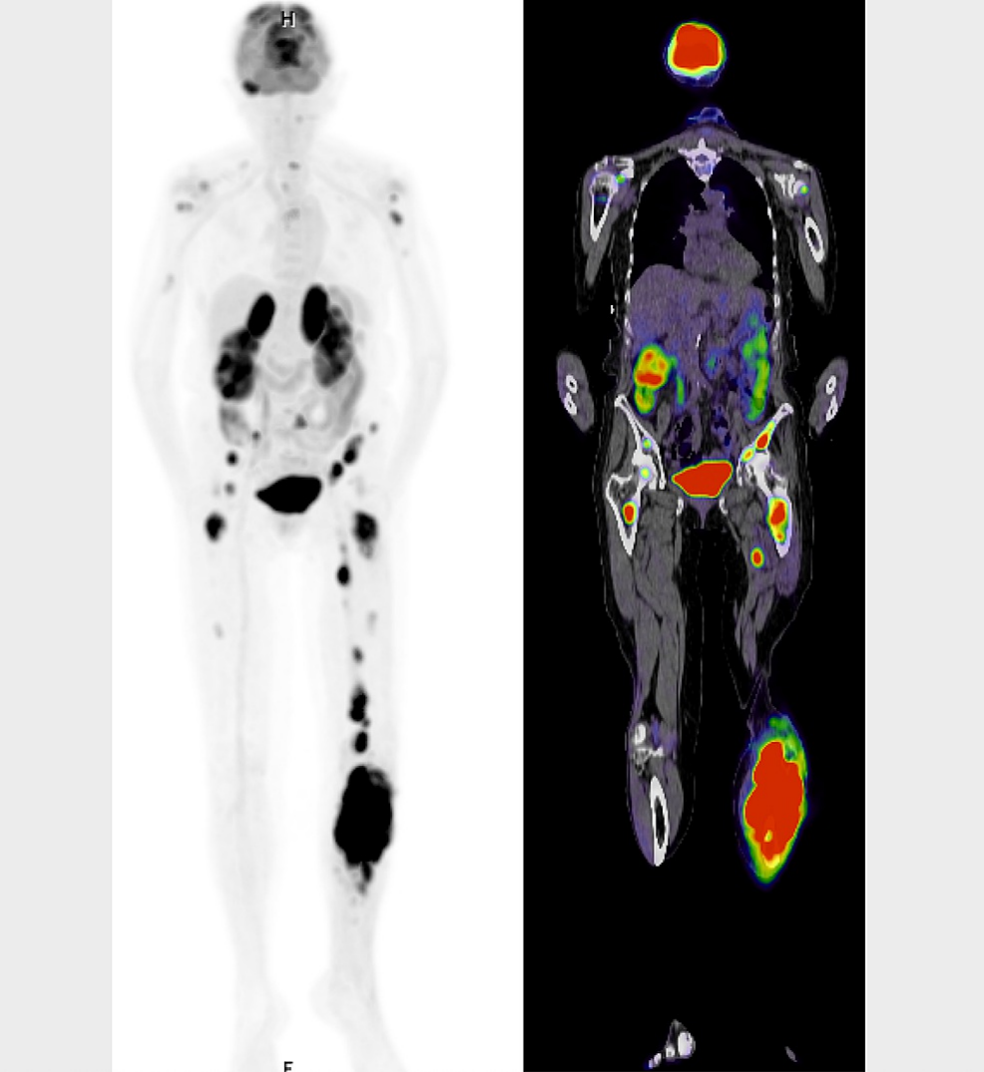
Positron emission tomography-computed tomography (PET-CT) scan demonstrating increased uptake in many areas, most notably the left tibia, which had a maximal standard uptake value (SUV) of 22.38. Other areas with high SUV values included the left proximal humerus (SUV 6.96), right temporal bone (SUV 9.41), left acetabulum (SUV 13.62), left adrenal gland (SUV 14.4), and the left adductor musculature (SUV 17.37). There is also multifocal lymphomatous involvement of the axial and appendicular skeleton.

CT of the chest, abdomen, and pelvis showed multiple inguinal and retroperitoneal lymph nodes and enlargement of the bilateral kidneys and adrenal glands. Core needle biopsy of the tibial mass revealed aggressive large B-cell lymphoma. Cells were CD20-positive and had a KI-67 proliferation rate of 70%. Immunohistochemical studies showed cells were reactive for CD10 and Bcl-6. C-myc and BCl-2 were overexpressed. Positron emission tomography scan showed widespread cancerous involvement of the axial and appendicular skeletons, adrenal glands, and kidneys (Figure 3). Lumbar puncture was negative for cerebral spinal fluid involvement.

The patient received six cycles of dose-adjusted rituximab, etoposide phosphate, prednisone, vincristine sulfate, cyclophosphamide, and doxorubicin hydrochloride. Her tibial fracture was treated with internal fixation and placement of an intramedullary rod. Six months after diagnosis, the patient is in clinical remission and doing well. She is now ambulating without difficulty.

## Discussion

Proximal tibial fracture is a rare presentation lymphoma [3]. Pathological fractures are much more frequently associated with metastatic lung, breast, thyroid, renal, prostate cancer, or primary bone sarcoma [4]. Among all cancers, the proximal femur, humerus, and pelvis are the most common sites of skeletal metastases [4]. When skeletal involvement is present in lymphoma, it is rarely found in the tibia; in a review of 131 patients with lymphoma involving the bone, only one patient had tibial involvement at diagnosis [5].

Upon initial presentation, our patient was discharged from the emergency room without having received a thorough workup for underlying malignancy and the diagnosis of lymphoma was missed. Although the diagnosis of lymphoma was unusual, it could have been made had the pathological fracture been worked up with appropriate history, physical examination, and imaging modalities. When a low-impact mechanism of injury is present or a destructive bone lesion is visible on plain radiograph, history should include personal history of cancer, family history of cancer, unintended weight loss, fevers, and antecedent pain [6]. Patients with pathological fractures should receive a thorough physical examination that includes lymph node palpation and evaluation for hepatosplenomegaly [7,8].

Radiographic workup should include CT scans of the chest, abdomen, and pelvis. MRI may be useful to distinguish bone metastases from osteoporosis [9]. While radiographic workup may help identify a primary tumor causing bony metastasis, it may also be negative; one study found that among patients with metastatic bone disease, primary disease site was identified on CT scan in only 28% of cases [10]. When a destructive bone lesion is present, underlying malignancy should not be ruled out based on CT alone; negative radiographic workup should be followed by bone biopsy [2].

Physicians should be aware that pathological fracture may be the presenting sign of lymphoma and include it on their differential when evaluating patients presenting with pathological fractures. Ultimately, our patient was diagnosed correctly and received the appropriate therapy. However, a significant amount of morbidity would likely have been avoided had a thorough workup for underlying malignancy been pursued. Our case highlights the importance of obtaining a thorough history of all patients who present with a low-energy fracture.

## Conclusions

Although lung, breast, thyroid, prostate, and renal metastases are more common, lymphoma should be considered on the differential of metastatic pathological fractures. While these fractures more commonly occur in the spine, femur, humerus, and pelvis, they can also occur in the tibia. Our case highlights the importance of careful examination of all patients who present with a low-energy fracture.
